# Crystal structure and spectroscopic properties of (*E*)-1,3-dimethyl-2-[3-(4-nitro­phen­yl)triaz-2-enyl­idene]-2,3-di­hydro-1*H*-imidazole

**DOI:** 10.1107/S2056989021000426

**Published:** 2021-01-15

**Authors:** Hector Mario Heras Martinez, David Chavez Flores, Patrick C. Hillesheim, Siddappa Patil, Alejandro Bugarin

**Affiliations:** a Department of Chemistry & Physics, Florida Gulf Coast University, 10501 FGCU, Boulevard South, Fort Myers, FL 33965, USA; b Facultad de Ciencias Químicas, Universidad Autónoma de Chihuahua, Nuevo Campus Universitario, Circuito Universitario, Chihuahua, Chih., CP 31125, Mexico; c Department of Chemistry and Physics, Ave Maria University, 5050 Ave Maria Blvd, Ave Maria, FL 34142, USA; d Centre for Nano and Material Sciences, Jain University, Jain Global Campus, Kanakapura, Ramanagaram, Bangalore 562112, India

**Keywords:** crystal structure, azides, π-conjugated triazenes, *N*-heterocyclic carbene

## Abstract

The title compound (*E*)-1,3-dimethyl-2-[3-(4-nitro­phen­yl)triaz-2-enyl­idene]-2,3-di­hydro-1*H*-imidazole, C_11_H_12_N_6_O_2_, has monoclinic (*C*2/*c*) symmetry at 100 K. This triazene derivative was synthesized by the coupling reaction of 1,3-di­methyl­imidazolium iodide with 1-azido-4-nitro benzene in the presence of sodium hydride (60% in mineral oil) and characterized by ^1^H NMR, ^13^C NMR, IR, mass spectrometry, and single-crystal X-ray diffraction. The mol­ecule consists of six-membered and five-membered rings, which are connected by a triazene moiety (–N=N—N–). In the solid-state, the mol­ecule is found to be planar due to conjugation throughout the mol­ecule. The extended structure shows two layers of mol­ecules, which present weak inter­molecular inter­actions that facilitate the stacked arrangement of the mol­ecules forming the extended structure. Furthermore, there are several weak pseudo-cyclical inter­actions between the nitro oxygen atoms and symmetry-adjacent H atoms, which help to arrange the mol­ecules.

## Abstract 2

The triazene derivative, (*E*)-1,3-dimethyl-2-[(4-nitro­phen­yl)triaz-2-enyl­idene]-2,3-di­hydro-1*H*-imidazole was synthesized by coupling 1,3-di­methyl­imidazolium iodide with 1-azido-4-nitro benzene. The title compound has monoclinic (*C*2/*c*) symmetry and an *E* conformation about the –N= N– bond.

## Chemical context   

Triazenes are versatile compounds in preparative chemistry because of their stable and highly modular nature (Patil & Bugarin, 2016 ▸). Triazene derivatives have been studied for their potential anti­cancer properties (Rouzer *et al.*, 1996 ▸; Connors *et al.*, 1976 ▸), used as a protecting group in natural product synthesis (Nicolaou *et al.*, 1999 ▸) and combinatorial chemistry (Brase *et al.*, 2000 ▸), incorporated into polymers (Jones *et al.*, 1997 ▸) and oligomer synthesis (Moore, 1997 ▸), and used to prepare heterocycles (Wirschun *et al.*, 1998 ▸). Their modular nature allows triazenes to be converted into a different functional group after treatment with the appropriate reagents. For example, aryl triazenes can be transformed to useful cross-coupling reagents (iodo­arenes) *via* iodo­methane-induced decomposition (Zollinger, 1994 ▸). Further, aryl triazenes have been studied for their unique structural and chemical properties (Cornali *et al.*, 2016 ▸; Knyazeva *et al.*, 2017 ▸). They have been used in medicinal, combinatorial chemistry, in natural synthesis and as organometallic ligands (Kimball *et al.*, 2002 ▸). In chemical biology, masked diazo­nium ions (tri­aza­butadienes) have found use on protein surfaces for identifying host proteins that inter­act during early stages of viral entities (Jensen *et al.*, 2016 ▸; Shadmehr *et al.*, 2018 ▸). In addition, tri­aza­butadienes have shown tunable reactivity under specific conditions. For example, unique transformations of tri­aza­butadienes have been affected *via* water solubility, pH (Kimani & Jewett, 2015 ▸; Guzman *et al.*, 2016 ▸; He *et al.*, 2017 ▸), and photoinduced isomerization (He *et al.*, 2015 ▸). In synthesis, these triazenes have been used as starting materials for aldehydes, ketones, ethers, and sulfides, under mild reaction conditions (Barragan & Bugarin, 2017 ▸; Cornali *et al.*, 2016 ▸). Furthermore, natural sunlight has been utilized to activate those triazenes to produce bis­aryls and anilides (Noonikara-Poyil *et al.*, 2019 ▸; Barragan *et al.*, 2020 ▸).
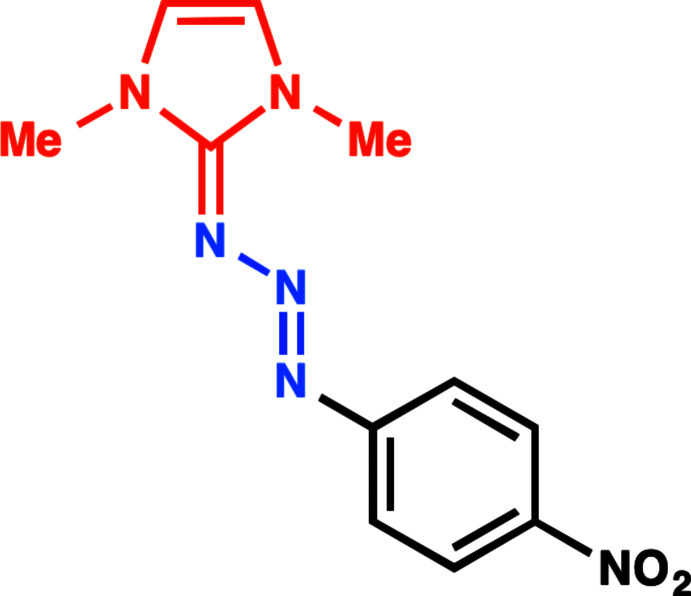


## Structural commentary   

**Figure 1:**
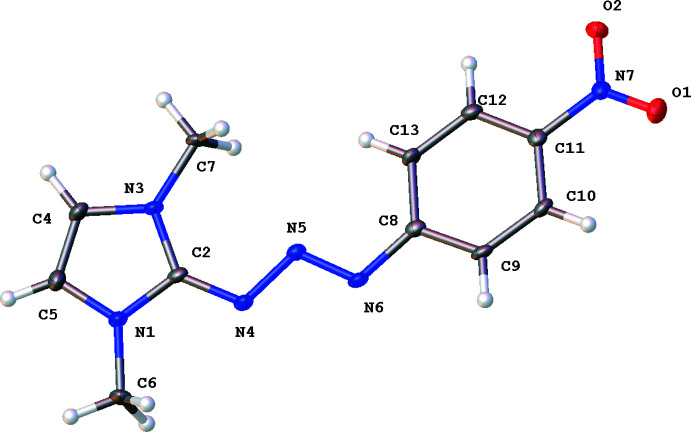
*ORTEP* diagram of compound **1** showing the atom-labeling scheme with 50% probability displacement ellipsoids.

The title compound **1** crystallizes in the monoclinic *C*2/*c* space group with a single moiety in the asymmetric unit (see Fig. 1 ▸). The mol­ecule is nearly planar with a dihedral angle of 7.36 (9)° between the imidazole and benzene rings. Plane **I** (N1/N3/C2/C4–C7) makes dihedral angles of 9.70 (3) and 2.40 (6)**°**, respectively, with planes **II** (N7/C8–C13) and **III** (N4–N6) while plane **II** makes a dihedral angle of 7.36 (4)**°** with plane **III**.

There are no lattice-held water mol­ecules or organic solvent mol­ecules in the unit cell of the determined structure, a potential issue since the starting material 1,3-di­methyl­imidazolium iodide is hydro­scopic. The bond lengths and angles in **1** are similar to those reported for analogous structures (Khramov & Bielawski, 2005 ▸; Jishkariani *et al.*, 2013 ▸). The C—C bond lengths in the phenyl rings are in the normal range of 1.33–1.40 Å, which is characteristic of delocalized phenyl rings. The C—C—C bond angles are around 120**°**, with the variation being less than 2**°**, which is characteristic of *sp* ^2^-hybridized carbons.

## Supra­molecular features   

**Figure 2:**
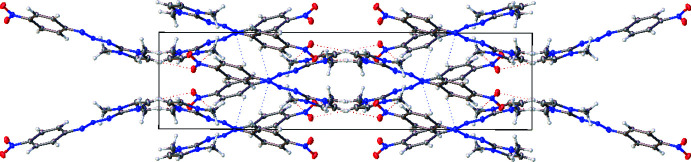
Perspective view of the mol­ecular packing of **1** (there are no hydrogen atoms involved in hydrogen-bonding inter­actions. Dotted lines represent weak non-covalent C—H⋯N and C—H⋯O interactions, which direct the packing.

Figs. 2 ▸ and 3 ▸ show a perspective view of the crystal packing of the title compound. The packing diagram shows two layers of mol­ecules, which are independently arranged in the unit cell without intra- and inter mol­ecular hydrogen bonds. In each layer, the mol­ecules are alternately parallel.

## Database survey   

The first X-ray structure of a π-conjugated triazene was reported by Khramov & Bielawski (2005 ▸). The current WebCSD structural database includes the structures of only 18 π-conjugated triazenes. However, there is only one structure, reported by our research group (Patil & Bugarin, 2014 ▸), that utilizes the small mol­ecule (1,3-di­methyl­imidazolium iodide) as the carbene coupling partner, and one more that bears an electron-deficient aryl group in combination with the small carbene precursor (Patil *et al.*, 2014 ▸). Those characteristics highlight the novelty and uniqueness of the compound reported herein.

## Synthesis and crystallization   

1-Azido-4-nitro benzene (Siddiki *et al.*, 2013 ▸) and 1,3-di­methyl­imidazolium iodide (Oertel *et al.*, 2011 ▸) were prepared according to literature procedures. For the synthesis of the title compound, 1-azido-4-nitro benzene (131.2 mg, 0.8 mmol) and 1,3-di­methyl­imidazolium iodide (89.5 mg, 0.4 mmol) were stirred at room temperature for 5 min. in dry THF (5 mL). NaH (16 mg, 0.4 mmol, 60% in mineral oil) was added in one portion and stirring was continued at room temperature overnight. A red precipitate formed, which was collected by filtration and dried under reduced pressure, giving the pure product (*E*)-1,3-dimethyl-2-[(4-nitro­phen­yl)triaz-2-enyl­idene])-2,3-di­hydro-1*H*-imidazole as a red crystalline solid (88.2 mg, 85%). Crystals suitable for X-ray analysis were grown from the slow evaporation of a THF/hexane mixture yielding air-stable, red-colored crystals.

IR (neat) ν 3435, 1572, 1382, 1360, 1233 cm^−1. 1^H NMR (400 MHz, DMSO-*d*6): δ 8.12 (*d*, *J* = 9.2 Hz, 2 H, Ph-H), 7.43 (*d*, *J* = 9.2 Hz, 2 H, Ph-H), 7.13 (*s*, 2 H, NCH), 3.62 (*s*, 6 H, N—CH_3_). ^13^C NMR (100 MHz, DMSO-*d*6): δ 159.1, 150.7, 143.2, 125.5, 120.8, 118.9, 35.7. UV/Vis (0.1 µ*M*, CH_2_Cl_2_): *λ* (*ɛ*) = 450 nm. HRMS (ESI, N_2_): *m*/*z* calculated for C_11_H_12_N_6_O_2_Na [*M* + Na]^+^ 283.0914, found 283.0918.

## Refinement   

Crystal data, data collection and structure refinement details are summarized in Table 1 ▸. H atoms were included in calculated positions and treated as riding atoms: C—H = 0.95–0.98 Å with *U*_iso_(H) = 1.2*U*_eq_(C) or 1.5*U*_eq_(C-meth­yl).

## Supplementary Material

Crystal structure: contains datablock(s) I. DOI: 10.1107/S2056989021000426/jy2004sup1.cif

Structure factors: contains datablock(s) I. DOI: 10.1107/S2056989021000426/jy2004Isup2.hkl

Supporting information file. DOI: 10.1107/S2056989021000426/jy2004Isup4.cdx

Supporting information file. DOI: 10.1107/S2056989021000426/jy2004Isup4.cml

CCDC reference: 2055595

Additional supporting information:  crystallographic information; 3D view; checkCIF report

## Figures and Tables

**Figure 3:**
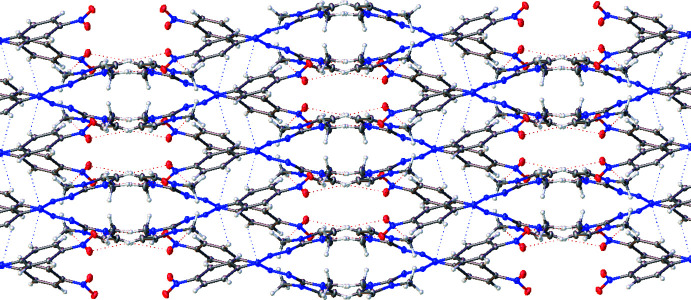
Extended network of the structure of compound **1** viewed from (001). There are two layers of mol­ecules, which are arranged independently in the unit cell without intra- and/or inter­molecular hydrogen bonds. Dotted lines represent weak non-covalent C–H⋯N and C–H⋯O interactions, which direct the packing.

**Table 1:** Experimental details

Crystal data	
Chemical formula	C_11_H_12_N_6_O_2_
*M*_r_	260.27
Crystal system, space group	Monoclinic, *C*2/*c*
Temperature (K)	100
*a*, *b*, *c* (Å)	27.7311 (18), 7.1747 (9), 11.7849 (14)
β (°)	94.101 (4)
*V* (Å^3^)	2338.7 (4)
*Z*	8
Radiation type	Mo *K*α
μ (mm^−1^)	0.11
Crystal size (mm)	0.11 × 0.05 × 0.02

Data collection	
Diffractometer	Bruker AXS D8 Quest diffractometer with PhotonII charge-integrating pixel array detector (CPAD)
Absorption correction	Multi-scan (*SADABS*; Bruker, 2018 ▸)
*T*_min_, *T*_max_	0.655, 0.746
No. of measured, independent and observed [*I* > 2σ(*I*)] reflections	23709, 2807, 2206
*R*_int_	0.077
(sin θ/λ)_max_ (Å^−1^)	0.659

Refinement	
*R*[*F* ^2^ > 2σ(*F* ^2^)], *wR*(*F* ^2^), *S*	0.070, 0.158, 1.16
No. of reflections	2807
No. of parameters	174
H-atom treatment	H-atom parameters constrained
Δρ_max_, Δρ_min_ (e Å^−3^)	0.36, −0.37

Computer programs: *APEX3* and *SAINT* (Bruker, 2018 ▸), *SHELXT2018*/2 (Sheldrick, 2015*a* ▸), *SHELXL* (Sheldrick, 2015*b* ▸) and *OLEX2* (Dolomanov *et al.*, 2009 ▸).

## supplementary crystallographic information

### Crystal data

**Table:**

C_11_H_12_N_6_O_2_	*F*(000) = 1088
*M**_r_* = 260.27	*D*_x_ = 1.478 Mg m^−^^3^
Monoclinic, *C*2/*c*	Mo *K*α radiation, λ = 0.71073 Å
*a* = 27.7311 (18) Å	Cell parameters from 4851 reflections
*b* = 7.1747 (9) Å	θ = 3.0–27.8°
*c* = 11.7849 (14) Å	µ = 0.11 mm^−^^1^
β = 94.101 (4)°	*T* = 100 K
*V* = 2338.7 (4) Å^3^	Plates, red
*Z* = 8	0.11 × 0.05 × 0.02 mm

### Data collection

**Table:**

Bruker AXS D8 Quest diffractometer with PhotonII charge-integrating pixel array detector (CPAD)	2206 reflections with *I* > 2σ(*I*)
φ and ω scans	*R*_int_ = 0.077
Absorption correction: multi-scan (SADABS; Bruker, 2018)	θ_max_ = 27.9°, θ_min_ = 2.9°
*T*_min_ = 0.655, *T*_max_ = 0.746	*h* = −36→36
23709 measured reflections	*k* = −9→9
2807 independent reflections	*l* = −15→15

### Refinement

**Table:**

Refinement on *F*^2^	0 restraints
Least-squares matrix: full	Hydrogen site location: inferred from neighbouring sites
*R*[*F*^2^ > 2σ(*F*^2^)] = 0.070	H-atom parameters constrained
*wR*(*F*^2^) = 0.158	*w* = 1/[σ^2^(*F*_o_^2^) + (0.0623*P*)^2^ + 4.2467*P*] where *P* = (*F*_o_^2^ + 2*F*_c_^2^)/3
*S* = 1.16	(Δ/σ)_max_ < 0.001
2807 reflections	Δρ_max_ = 0.36 e Å^−^^3^
174 parameters	Δρ_min_ = −0.37 e Å^−^^3^

### Special details

**Table:**

Geometry. All esds (except the esd in the dihedral angle between two l.s. planes) are estimated using the full covariance matrix. The cell esds are taken into account individually in the estimation of esds in distances, angles and torsion angles; correlations between esds in cell parameters are only used when they are defined by crystal symmetry. An approximate (isotropic) treatment of cell esds is used for estimating esds involving l.s. planes.

### Fractional atomic coordinates and isotropic or equivalent isotropic displacement parameters (Å^2^)

**Table:**

	*x*	*y*	*z*	*U*_iso_*/*U*_eq_	
O2	0.90725 (6)	0.6192 (3)	0.42545 (14)	0.0208 (4)	
O1	0.91792 (6)	0.7705 (3)	0.58417 (15)	0.0231 (4)	
N5	0.67583 (7)	0.4345 (3)	0.54983 (15)	0.0122 (4)	
N3	0.60385 (7)	0.2903 (3)	0.39246 (15)	0.0133 (4)	
N6	0.70494 (7)	0.4980 (3)	0.63041 (15)	0.0144 (4)	
N1	0.55532 (7)	0.2913 (3)	0.52968 (16)	0.0163 (4)	
N7	0.89267 (7)	0.6760 (3)	0.51636 (16)	0.0156 (4)	
N4	0.63305 (7)	0.3932 (3)	0.58753 (15)	0.0147 (4)	
C2	0.60118 (8)	0.3293 (3)	0.50481 (18)	0.0132 (5)	
C8	0.75071 (8)	0.5395 (3)	0.59381 (18)	0.0131 (5)	
C13	0.76739 (8)	0.4927 (3)	0.48705 (19)	0.0138 (5)	
H13	0.746476	0.430092	0.432205	0.017*	
C10	0.82858 (8)	0.6785 (3)	0.64889 (18)	0.0140 (5)	
H10	0.849717	0.740951	0.703362	0.017*	
C12	0.81359 (8)	0.5371 (3)	0.46184 (19)	0.0150 (5)	
H12	0.824761	0.505139	0.390018	0.018*	
C9	0.78212 (8)	0.6344 (3)	0.67289 (18)	0.0140 (5)	
H9	0.771038	0.668868	0.744390	0.017*	
C11	0.84393 (8)	0.6295 (3)	0.54293 (19)	0.0148 (5)	
C7	0.64554 (8)	0.3132 (3)	0.32329 (18)	0.0157 (5)	
H7A	0.656635	0.442877	0.327693	0.023*	
H7B	0.635989	0.281514	0.243959	0.023*	
H7C	0.671761	0.230600	0.352109	0.023*	
C4	0.55897 (8)	0.2268 (4)	0.34928 (19)	0.0184 (5)	
H4	0.550771	0.189696	0.272952	0.022*	
C5	0.52938 (8)	0.2270 (4)	0.4335 (2)	0.0200 (5)	
H5	0.496428	0.189691	0.428140	0.024*	
C6	0.53830 (9)	0.3056 (4)	0.6434 (2)	0.0249 (6)	
H6A	0.553996	0.209729	0.692393	0.037*	
H6B	0.503190	0.287618	0.639429	0.037*	
H6C	0.546263	0.429166	0.674856	0.037*	

### Atomic displacement parameters (Å^2^)

**Table:**

	*U*^11^	*U*^22^	*U*^33^	*U*^12^	*U*^13^	*U*^23^
O2	0.0169 (8)	0.0327 (10)	0.0134 (8)	−0.0025 (7)	0.0046 (6)	0.0010 (8)
O1	0.0202 (9)	0.0287 (10)	0.0195 (9)	−0.0084 (8)	−0.0042 (7)	−0.0001 (8)
N5	0.0143 (9)	0.0132 (9)	0.0092 (8)	0.0006 (7)	0.0006 (7)	0.0000 (7)
N3	0.0145 (9)	0.0185 (10)	0.0072 (8)	−0.0003 (8)	0.0022 (7)	0.0002 (7)
N6	0.0159 (10)	0.0204 (10)	0.0070 (8)	0.0011 (8)	0.0010 (7)	−0.0003 (8)
N1	0.0133 (9)	0.0269 (11)	0.0089 (9)	−0.0009 (8)	0.0009 (7)	0.0009 (8)
N7	0.0147 (9)	0.0192 (10)	0.0126 (9)	−0.0016 (8)	−0.0004 (7)	0.0040 (8)
N4	0.0136 (9)	0.0226 (10)	0.0080 (8)	0.0006 (8)	0.0013 (7)	−0.0019 (8)
C2	0.0158 (11)	0.0162 (11)	0.0076 (10)	0.0026 (9)	0.0002 (8)	0.0020 (8)
C8	0.0161 (11)	0.0134 (10)	0.0097 (10)	0.0022 (9)	0.0002 (8)	0.0015 (8)
C13	0.0149 (11)	0.0172 (11)	0.0090 (10)	−0.0015 (9)	−0.0004 (8)	−0.0032 (8)
C10	0.0174 (11)	0.0156 (11)	0.0084 (10)	0.0001 (9)	−0.0035 (8)	0.0003 (8)
C12	0.0180 (11)	0.0183 (11)	0.0088 (10)	0.0005 (9)	0.0014 (8)	0.0017 (9)
C9	0.0184 (11)	0.0177 (11)	0.0059 (9)	0.0032 (9)	0.0003 (8)	0.0008 (9)
C11	0.0146 (11)	0.0179 (11)	0.0118 (10)	−0.0005 (9)	−0.0007 (8)	0.0052 (9)
C7	0.0164 (11)	0.0238 (12)	0.0073 (10)	−0.0006 (9)	0.0047 (8)	−0.0004 (9)
C4	0.0171 (11)	0.0281 (13)	0.0096 (10)	−0.0009 (10)	−0.0024 (8)	−0.0011 (10)
C5	0.0146 (11)	0.0308 (14)	0.0141 (11)	−0.0040 (10)	−0.0032 (9)	0.0027 (10)
C6	0.0169 (11)	0.0473 (17)	0.0112 (11)	−0.0049 (12)	0.0063 (9)	−0.0017 (11)

### Geometric parameters (Å, º)

**Table:**

O2—N7	1.241 (3)	C13—C12	1.373 (3)
O1—N7	1.228 (3)	C10—H10	0.9500
N5—N6	1.285 (3)	C10—C9	1.375 (3)
N5—N4	1.330 (3)	C10—C11	1.393 (3)
N3—C2	1.360 (3)	C12—H12	0.9500
N3—C7	1.471 (3)	C12—C11	1.395 (3)
N3—C4	1.387 (3)	C9—H9	0.9500
N6—C8	1.402 (3)	C7—H7A	0.9800
N1—C2	1.353 (3)	C7—H7B	0.9800
N1—C5	1.378 (3)	C7—H7C	0.9800
N1—C6	1.456 (3)	C4—H4	0.9500
N7—C11	1.448 (3)	C4—C5	1.332 (3)
N4—C2	1.348 (3)	C5—H5	0.9500
C8—C13	1.412 (3)	C6—H6A	0.9800
C8—C9	1.405 (3)	C6—H6B	0.9800
C13—H13	0.9500	C6—H6C	0.9800
			
N6—N5—N4	111.13 (17)	C11—C12—H12	120.4
C2—N3—C7	128.02 (19)	C8—C9—H9	119.3
C2—N3—C4	108.35 (18)	C10—C9—C8	121.4 (2)
C4—N3—C7	123.61 (18)	C10—C9—H9	119.3
N5—N6—C8	112.51 (18)	C10—C11—N7	119.1 (2)
C2—N1—C5	109.43 (19)	C10—C11—C12	121.7 (2)
C2—N1—C6	123.82 (19)	C12—C11—N7	119.2 (2)
C5—N1—C6	126.6 (2)	N3—C7—H7A	109.5
O2—N7—C11	118.54 (19)	N3—C7—H7B	109.5
O1—N7—O2	122.5 (2)	N3—C7—H7C	109.5
O1—N7—C11	118.95 (19)	H7A—C7—H7B	109.5
N5—N4—C2	112.85 (18)	H7A—C7—H7C	109.5
N1—C2—N3	106.65 (19)	H7B—C7—H7C	109.5
N4—C2—N3	134.0 (2)	N3—C4—H4	125.9
N4—C2—N1	119.30 (19)	C5—C4—N3	108.1 (2)
N6—C8—C13	125.8 (2)	C5—C4—H4	125.9
N6—C8—C9	115.53 (19)	N1—C5—H5	126.3
C9—C8—C13	118.6 (2)	C4—C5—N1	107.5 (2)
C8—C13—H13	119.7	C4—C5—H5	126.3
C12—C13—C8	120.5 (2)	N1—C6—H6A	109.5
C12—C13—H13	119.7	N1—C6—H6B	109.5
C9—C10—H10	120.7	N1—C6—H6C	109.5
C9—C10—C11	118.5 (2)	H6A—C6—H6B	109.5
C11—C10—H10	120.7	H6A—C6—H6C	109.5
C13—C12—H12	120.4	H6B—C6—H6C	109.5
C13—C12—C11	119.2 (2)		
			
O2—N7—C11—C10	174.5 (2)	C13—C12—C11—N7	179.9 (2)
O2—N7—C11—C12	−5.6 (3)	C13—C12—C11—C10	−0.1 (3)
O1—N7—C11—C10	−5.8 (3)	C9—C8—C13—C12	0.9 (3)
O1—N7—C11—C12	174.2 (2)	C9—C10—C11—N7	179.7 (2)
N5—N6—C8—C13	−9.6 (3)	C9—C10—C11—C12	−0.3 (3)
N5—N6—C8—C9	171.13 (19)	C11—C10—C9—C8	1.0 (3)
N5—N4—C2—N3	3.5 (4)	C7—N3—C2—N1	178.1 (2)
N5—N4—C2—N1	−176.5 (2)	C7—N3—C2—N4	−1.8 (4)
N3—C4—C5—N1	0.3 (3)	C7—N3—C4—C5	−178.5 (2)
N6—N5—N4—C2	178.84 (19)	C4—N3—C2—N1	−0.3 (3)
N6—C8—C13—C12	−178.4 (2)	C4—N3—C2—N4	179.8 (3)
N6—C8—C9—C10	178.0 (2)	C5—N1—C2—N3	0.5 (3)
N4—N5—N6—C8	178.84 (18)	C5—N1—C2—N4	−179.6 (2)
C2—N3—C4—C5	0.0 (3)	C6—N1—C2—N3	176.7 (2)
C2—N1—C5—C4	−0.5 (3)	C6—N1—C2—N4	−3.4 (4)
C8—C13—C12—C11	−0.2 (3)	C6—N1—C5—C4	−176.5 (2)
C13—C8—C9—C10	−1.3 (3)		

## References

[bb1] Barragan, E. & Bugarin, A. (2017). *J. Org. Chem.* **82**, 1499–1506. 10.1021/acs.joc.6b02705 28085277

[bb2] Barragan, E., Noonikara–Poyil, A. & Bugarin, A. (2020). *Asia. J. Org. Chem.* **9**, 445–445.

[bb3] Bräse, S., Dahmen, S. & Pfefferkorn, M. (2000). *J. Comb. Chem.* **2**, 710–715. 10.1021/cc000051s 11126299

[bb4] Bruker (2018). *APEX3*, *SAINT* and *SADABS*. Bruker AXS Inc., Madison, Wisconsin, USA.

[bb5] Connors, T. A., Goddard, P. M., Merai, K., Ross, W. C. J. & Wilman, D. E. V. (1976). *Biochem. Pharmacol.* **25**, 241–246. 10.1016/0006-2952(76)90207-0 1267820

[bb6] Cornali, B. M., Kimani, F. W. & Jewett, J. C. (2016). *Org. Lett.* **18**, 4948–4950. 10.1021/acs.orglett.6b02420 27619479

[bb7] Dolomanov, O. V., Bourhis, L. J., Gildea, R. J., Howard, J. A. K. & Puschmann, H. (2009). *J. Appl. Cryst.* **42**, 339–341.

[bb8] Guzman, L. E., Kimani, F. W. & Jewett, J. C. (2016). *ChemBioChem*, **17**, 2220–2222. 10.1002/cbic.201600517 PMC5170869 27662242

[bb9] He, J., Kimani, F. W. & Jewett, J. C. (2015). *J. Am. Chem. Soc.* **137**, 9764–9767. 10.1021/jacs.5b04367 26214020

[bb10] He, J., Kimani, F. W. & Jewett, J. C. (2017). *Synlett*, **28**, 1767–1770.

[bb11] Jensen, S. M., Kimani, F. W. & Jewett, J. C. (2016). *ChemBioChem*, **17**, 2216–2219. 10.1002/cbic.201600508 PMC5170875 27647786

[bb12] Jishkariani, D., Hall, C. D., Demircan, A., Tomlin, B. J., Steel, P. J. & Katritzky, A. R. (2013). *J. Org. Chem.* **78**, 3349–3354. 10.1021/jo302697q 23390958

[bb13] Jones, L. R., Schumm, J. S. & Tour, J. M. (1997). *J. Org. Chem.* **62**, 1388–1410.

[bb14] Khramov, D. M. & Bielawski, C. W. (2005). *Chem. Commun.* pp. 4958–4960. 10.1039/b508679e 16205813

[bb15] Kimani, F. W. & Jewett, J. C. (2015). *Angew. Chem. Int. Ed.* **54**, 4051–4054. 10.1002/anie.201411277 25663253

[bb16] Kimball, D. B., Herges, R. & Haley, M. M. (2002). *J. Am. Chem. Soc.* **124**, 1572–1573. 10.1021/ja017227u 11853420

[bb17] Knyazeva, D. C., Kimani, F. W., Blanche, J. L. & Jewett, J. C. (2017). *Tetrahedron Lett.* **58**, 2700–2702.

[bb18] Moore, J. S. (1997). *Acc. Chem. Res.* **30**, 402–413.

[bb19] Nicolaou, K. C., Boddy, C. N. C., Li, H., Koumbis, A. E., Hughes, R., Natarajan, S., Jain, N. F., Ramanjulu, J. M., Bräse, S. & Solomon, M. E. (1999). *Chem. Eur. J.* **5**, 2602–2621.

[bb20] Noonikara-Poyil, A., Barragan, E., Patil, S. & Bugarin, A. (2019). *J. Mex. Chem. Soc.* **63**, 84–92.

[bb21] Oertel, A. M., Ritleng, V., Burr, L. & Chetcuti, M. J. (2011). *Organometallics*, **30**, 6685–6691.

[bb22] Patil, S. & Bugarin, A. (2014). *Acta Cryst.* E**70**, 224–227. 10.1107/S1600536814020698 PMC4257158 25484658

[bb23] Patil, S. & Bugarin, A. (2016). *Eur. J. Org. Chem.* pp. 860–870.

[bb24] Patil, S., White, K. & Bugarin, A. (2014). *Tetrahedron Lett.* **55**, 4826–4829.

[bb25] Rouzer, C. A., Sabourin, M., Skinner, T. L., Thompson, E. J., Wood, T. O., Chmurny, G. N., Klose, J. R., Roman, J. M., Smith, R. H. & Michejda, C. J. (1996). *Chem. Res. Toxicol.* **9**, 172–178. 10.1021/tx9500639 8924588

[bb26] Shadmehr, M., Davis, G. J., Mehari, B. T., Jensen, S. M. & Jewett, J. C. (2018). *ChemBioChem*, **19**, 2550–2552. 10.1002/cbic.201800599 PMC6457986 30341988

[bb27] Sheldrick, G. M. (2015*a*). *Acta Cryst.* A**71**, 3–8.

[bb28] Sheldrick, G. M. (2015*b*). *Acta Cryst.* C**71**, 3–8.

[bb29] Siddiki, A. A., Takale, B. S. & Telvekar, V. N. (2013). *Tetrahedron Lett.* **54**, 1294–1297.

[bb30] Wirschun, W., Winkler, M., Lutz, K. & Jochims, J. C. (1998). *J. Chem. Soc. Perkin Trans. 1*, pp. 1755–1762.

[bb31] Zollinger, H. (1994). *Diazo Chemistry*, Vol I, pp. 382–404. Weinheim: VCH.

